# Outcomes of a state-wide virtual emergency department in Victoria, Australia: a linked data analysis of emergency department utilisation and hospital use

**DOI:** 10.1016/j.lanwpc.2026.101985

**Published:** 2026-09-19

**Authors:** Loren M. Sher, Rebecca L. Jessup, James H. Boyd, Jennifer Hutton, Mani Suleiman, Joanna Lawrence, Suzanne Miller, Ziad Nehme, David Bevan, Adam Ivan Semciw

**Affiliations:** a Victorian Virtual Emergency Department, The Northern Hospital, Northern Health, Melbourne, Victoria, Australia; b School of Psychology and Public Health, La Trobe University, Melbourne, Victoria, Australia; c Department of Critical Care, Melbourne Medical School, The University of Melbourne, Melbourne, Victoria, Australia; d Department of Medicine, Melbourne Medical School, The University of Melbourne, Melbourne, Victoria, Australia; e School of Allied Health, Human Services and Sport, La Trobe University, Melbourne, Victoria, Australia; f Victorian Centre for Virtual Health Research, Northern Health, Melbourne, Victoria, Australia; g Allied Health, Northern Health, Melbourne, Victoria, Australia; h Centre for Research and Evaluation, Ambulance Victoria, Blackburn North, Victoria, Australia; i School of Public Health and Preventive Medicine, Monash University, Melbourne, Victoria, Australia; j eHealth Division, Victorian Department of Health, Melbourne, Victoria, Australia; k Research Development and Governance Unit, Northern Health, Victoria, Australia; l Research and Industry Engagement, Latrobe University, Victoria, Australia

**Keywords:** Virtual Emergency Department, Telehealth, Hospital avoidance, Unplanned presentations

## Abstract

**Background:**

The Victorian Virtual Emergency Department (VVED) is a synchronous video-based emergency care service established to reduce the burden on physical EDs by managing patients remotely. Despite rapid growth in virtual emergency care models, evidence regarding downstream hospital utilisation and factors associated with subsequent ED presentation remains limited. We aimed to examine downstream ED utilisation following a VVED consultation and identify factors associated with unplanned ED presentation.

**Methods:**

We conducted a retrospective cohort study using linked statewide administrative data. The study included all patients registered with the VVED between 1 July 2022 and 23 June 2023. The primary outcome was unplanned ED presentation within 48 h among patients initially managed in the community. Secondary outcomes included discharge destination and planned and unplanned ED utilisation within 48 h and 7 days. Mixed-effects logistic regression was used to identify factors associated with unplanned ED presentation.

**Findings:**

Of 100,422 VVED presentations, 75.5% (n = 75,871) were managed at home and 15.3% (n = 15,353) were referred to a physical ED. Among patients managed at home, 8.2% (n = 6202) attended a physical ED within 48 h, increasing to 11.4% (n = 8667) within 7 days. Overall, 81.5% (n = 81,840) of patients did not attend a physical ED within 48 h. Primary diagnosis category was the only factor independently associated with unplanned ED presentation within 48 h.

**Interpretation:**

Most patients were managed in the community, with low unplanned ED utilisation. Higher unplanned rates in our Healthcare Professional pathway likely reflect greater clinical complexity. Further research is needed to explore long-term outcomes, patient perspectives, optimal referral pathways for high-risk groups and the temporal and causal relationship between VVED implementation and physical ED utilisation.

**Funding:**

This study was conducted as part of a Digital Health Cooperative Research Centre project, a partnership between La Trobe University, Northern Health, and the Victorian Department of Health.

**Research in context**

**Evidence before this study**

We searched MEDLINE, CINAHL, and EMBASE for studies published in the last 15 years (Jan 01,2011, to Dec 31, 2025) evaluating virtual or telehealth emergency department models and their downstream impact on physical ED use and hospital utilisation. Our search combined terms related to virtual care (telehealth, digital health, virtual consultation, virtual emergency department) with terms related to emergency system outcomes (ED presentation, hospital admission, diversion attendance, triage, and revisit). While virtual ED models have grown globally since the COVID-19 pandemic, most published studies are limited to pilot programmes or single-site implementations focusing on patient experience, triage accuracy, or call volumes. Few have assessed system-level outcomes at population scale, and no study had used linked administrative data to track patient care pathways following a virtual emergency consultation across an entire state.

**Added value of this study**

This is the first population-level, linked administrative data study to examine hospital utilisation following consultation with a state-wide virtual emergency department. Using linked data from 100,422 presentations to the Victorian Virtual Emergency Department (VVED) over its first full year of state-wide operation, we tracked downstream ED attendance and hospital admission across eight distinct referral pathways. Our findings demonstrated low rates of subsequent unplanned ED presentation following VVED consultation, comparable to or lower than rates reported after discharge from physical emergency departments and nurse-led telephone triage services internationally. By stratifying outcomes across referral pathways, we also provide new evidence on how clinical complexity influences hospital utilisation following virtual emergency care.

**Implications of all the available evidence**

These findings suggest that virtual emergency departments may be a scalable complement to traditional emergency services, with most patients not subsequently attending a physical ED and low rates of unplanned ED presentation. Our population-level evidence base supports investment in and integration of virtual emergency care for health systems facing unsustainable ED pressures. Policymakers should consider referral pathway design carefully for higher-risk groups, including frail older adults in residential aged care. Future research should examine longer-term outcomes including mortality, patient decision-making, and equity of access to ensure virtual emergency care remains safe, sustainable, and appropriately targeted.

## Introduction

Globally, emergency departments (EDs) are facing unprecedented and unsustainable levels of demand.^1^ This growing demand is driven by an ageing population, increasing prevalence of chronic and complex diseases, limited access to alternative care pathways (particularly after hours), and gaps in health system coordination.^2^ This demand contributes to longer wait times, overcrowding, and adverse patient outcomes, while placing considerable pressure on health professionals and hospital resources.^3^^,^^4^ Access block and patient dissatisfaction are widespread, raising questions about the sustainability of current emergency care models.^3^, ^4^, ^5^

Telehealth-based models of emergency care have emerged internationally as a promising strategy to manage ED demand. A recent scoping review of international evidence found that telehealth models have been used successfully to manage demand by improving triage, redirecting low-acuity cases, and enhancing access to timely care.^6^ These models encompass a spectrum of services, ranging from nurse-led advice lines and general practitioner (GP) teleconsultations to full virtual emergency departments. Collectively, these models aim to provide an alternative virtual care pathway while reducing unnecessary physical presentations to the ED.

The COVID-19 pandemic exacerbated existing pressures on EDs, disrupting routine services and necessitating rapid adaptation across health systems. Concerns over personal exposure to the virus, coupled with strict lockdown measures, deterred many from seeking timely emergency care.^7^ In addition, protecting healthcare workers from infection risk became a critical priority. Collectively, these challenges created an urgent need for innovative models of care. In October 2020, Northern Health (Melbourne, Australia) launched a Virtual Emergency Department (VED) to reduce pressure on its physical ED by providing timely, clinician-led care to patients with lower-acuity presentations who could safely remain at home.^8^^,^^9^ Initially, only serving the local catchment area, the model rapidly evolved in response to growing demand and demonstrated success, and by February 2022, the service became the Victorian Virtual Emergency Department (VVED) - a 24/7, state-wide service.^9^ It has since become one of the largest virtual emergency departments in the world, with more than 800,000 patient consultations to date (March 2026). During the study period (2022–2023), the VVED managed 100,422 presentations, representing approximately 5.3% of all Victorian public emergency department presentations reported to the Australin Institute of Health and Welfare (1,894,732) during the same period, highlighting the scale of the service within the state's emergency care system.^10^

Despite the rapid growth of virtual models like the VVED, evidence regarding their broader clinical and system-level impacts remains limited.^6^ To date, most evaluations have focused on patient and clinician experience or have been restricted to single-site audits of routinely collected administrative data. Such approaches provide limited insight into the pathways patients take following a virtual consultation or the extent to which these services successfully reduce immediate pressure on physical EDs.^11^, ^12^, ^13^, ^14^ Return visits to the ED are recognised as a marker of potential adverse outcomes, and monitoring these presentations is widely used as an indicator of ED quality.^15^^,^^16^ The objective of this study was to examine downstream outcomes following an index VVED presentation. Specifically, we aimed to:

i) Describe discharge destination following VVED presentation
ii) Describe downstream ED utilisation following a VVED presentation, including overall ED avoidance, and planned and unplanned ED presentations within 48 h and 7 days of an index VVED presentation; and
iii) Identify factors associated with unplanned ED presentations within 48 h among patients initially managed in the community following a VVED presentation.

## Methods

### Study design

We conducted a retrospective cohort study using routinely collected data for patients presenting to the VVED between 01 July 2022 and 23 June 2023 (the first financial year operating as a state-wide service), linked to the Victorian Emergency Minimum Dataset (VEMD) and the Victorian Admitted Episodes Dataset (VAED). The VEMD includes episode-level data on presentations to all public hospital emergency departments across Victoria, and the VAED contains data on all admitted patient episodes in public and private hospitals in Victoria.

The study has been reported according to the Reporting of studies Conducted using Observational Routinely-collected health Data (RECORD) Statement.^17^ This study was conducted in accordance with the Declaration of Helsinki. Ethics approval was obtained from St Vincent's Hospital Human Research Ethics Committee (Approval number 82094), which granted a waiver of informed consent given the study's use of routinely collected, subsequently deidentified data.

### Setting

The VVED is a state-wide telehealth service delivering real-time emergency care via video consultations.^9^ It enables patients to connect with emergency clinicians from home, work, or community settings for assessment, management, and appropriate disposition, reducing the need for physical ED attendance. The model is designed to improve access, reduce avoidable ED presentations, and support system capacity. VVED was developed and continues to be run by Northern Health, a major public health service in Melbourne’s North serving one of Victoria’s most diverse and rapidly growing populations. Northern Health has been at the forefront of digital innovation, developing and implementing multiple virtual and digitally enabled models of care across acute, emergency, cancer, and community settings.^18^, ^19^, ^20^, ^21^, ^22^ The service is delivered by emergency nurses, emergency physicians, nurse practitioners and general practitioners using synchronous video consultations.

The VVED operates across two core registration pathways. *Patient self-referral* allows individuals to register online via the VVED website, complete pre-screening questions, and enter a virtual triage queue where an emergency nurse conducts a video assessment before either providing self-care advice, referring to primary care, or escalating to a VVED doctor or nurse practitioner. *Health care provider* referral enables clinicians, including paramedics, GPs, urgent care centres and aged care staff to register patients through dedicated links that bypass nurse triage and connect directly to the clinician consult waiting room.

### Participants

Participants comprised all patients who completed registration with the VVED service between 1 July 2022 and 23 June 2023. The week of 23–30 June was used to follow up on presentation and hospital admission outcomes.

### Data sources, access and linkage

Presentation-level data for all ED presentations and hospital admissions across the state of Victoria, including both physical and virtual presentations, were obtained from the VEMD and the VAED. These standardised datasets are reported by all public hospitals in Victoria. A linked, de-identified data extract of all physical and virtual presentations during the study period was provided by the Centre for Victorian Data Linkage (CVDL) and stored on a secure remote server, accessible only to approved study investigators. All reported discharge destinations are derived from this linked dataset.

As Northern Health administered the VVED, its records were considered the definitive source for VVED-related activity. Unique Record Numbers (URNs) and presentation date and time for all VVED activity during the study period were extracted, imported into the secure remote server and linked to the VEMD dataset by a biostatistician. Observations that matched were classified as VVED presentations within the VEMD dataset; unmatched presentations were considered physical ED presentations.

### Outcomes

The primary outcome was the proportion of VVED consultations resulting in an unplanned ED presentation and/or hospital admission within 48 h of the index consultation, consistent with the Australian Council on Healthcare Standards benchmark.^15^ Secondary outcomes included ED presentation and/or hospital admission within 7 days. A 7-day follow-up window has been used in multiple studies examining ED return visits and virtual urgent care outcomes, enabling comparison with the existing literature.^13^^,^^23^

#### Variables

##### Discharge destination

We summarised discharged destination across four categories:

• Home: patients referred to community health providers (e.g., primary care, ambulatory care) or advised that no further care was required.
• ED: patients directed to a hospital ED via ambulance or private transport.
• Direct Admit: patients directly admitted to hospital.
• Other: patients who did not complete the VVED consultation, commonly due to technology issues or a change in their clinical condition.

##### Demographic variables

Patient demographic variables included gender (female, male, indeterminate, other), birth country (Australia, New Zealand, other), and English-speaking status (yes, no). Age was presented as both a continuous outcome (mean, SD) and categorised in ten-year increments.

##### Socio-economic factors

Socio-economic factors included socio-economic status (SES) and regional classification (metro, regional, rural/remote (Modified Monash (MM) model)). SES was obtained by matching residential postal area codes to the Index of Relative Socio-economic Advantage and Disadvantage (IRSAD) 2021 classifications provided by the Australian Bureau of Statistics,^24^ and presented in quintiles: most disadvantaged, disadvantaged, middle SES, advantaged, most advantaged. Regionality was obtained by matching participants’ residential suburb to the 2019 Modified Monash Model (MMM)^25^ classifications and consolidating these into metro (MM = 1), regional (MM = 2) and rural/remote (MM = 3–7).

Health service factors included registration source (self-referral, healthcare professional referral) and interpreter request status (yes, no).

Clinical characteristics were summarised using primary ICD-10-AM chapter-level groupings,^26^ including broad categories such as symptoms and abnormal findings, respiratory diseases, circulatory diseases, injury/poisoning, and mental health disorders.

### Data analysis

#### Discharge destination

A descriptive analysis was conducted to summarise patient presentations and discharge destination (home, emergency department, direct admission, or other), stratified by demographic variables, socio-economic factors, health service factors, and clinical characteristics.

#### Hospital ED avoidance

Overall, ED avoidance was defined as the percent of records that avoided hospital ED presentation within 48 h, regardless of discharge destination.

#### Planned and unplanned hospital utilisation (48 h, 7 days)

Subsequent hospital utilisation within 48 h and 7 days of the index presentation was summarised by counts and proportions of ED presentations and hospital admissions, stratified by discharge destination and registration source (self/healthcare professional). Unplanned ED presentations were defined as those discharged to Home who subsequently presented to a physical ED within 48 h or 7 days (Equation 1, Supplementary Methods). Planned ED presentations were defined as those referred to ED at the index consultation who subsequently attended a physical ED within 48 h or 7 days (Equation 2, Supplementary Methods).

#### Factors associated with unplanned ED presentations within 48 h of a VVED presentation

Mixed-effects univariable logistic regression models were used to assess the independent association between demographic, socio-economic, health service utilisation or clinical factors with physical ED hospital re-presentation within 48 h of an index VVED presentation. Independent variables were entered as fixed effects, while participant ID was entered as a random effect to account for repeat presentations by the same individual. Due to small counts and unstable parameter estimates, individuals with gender classified as ‘indeterminate’ or ‘other’, those referred through the Healthcare for the Homeless pathway, and presentations classified as Neoplasms (C00-D49) or ‘Congenital malformations, deformations and chromosomal abnormalities’ (Q00-Q99), were excluded from regression analyses.

Multivariable regression was then performed to assess the adjusted association between independent variables and the outcome. Variables associated with unplanned ED presentation at p < 0.20 in univariable analyses were considered for inclusion in the multivariable mixed-effects logistic regression model. Adjusted Odds Ratios (OR) with 95% confidence intervals (CI) were calculated to estimate the association between each variable and unplanned ED presentations in 48 h. Statistical significance was defined at p < 0.05. All analyses were conducted in R version 4.4.0 (R Foundation for Statistical Computing, Vienna, Austria).

### Role of the funding source

The funders had no involvement in study design, data collection, data analysis, data interpretation, or the writing of the report.

## Results

A total of 100,556 presentations were recorded over the study period. Of these, 135 (0.13%) presentations were unable to be joined to the linked dataset, leaving a final dataset of 100,422 records from 80,161 people (Fig. 1).

**Fig. 1:**
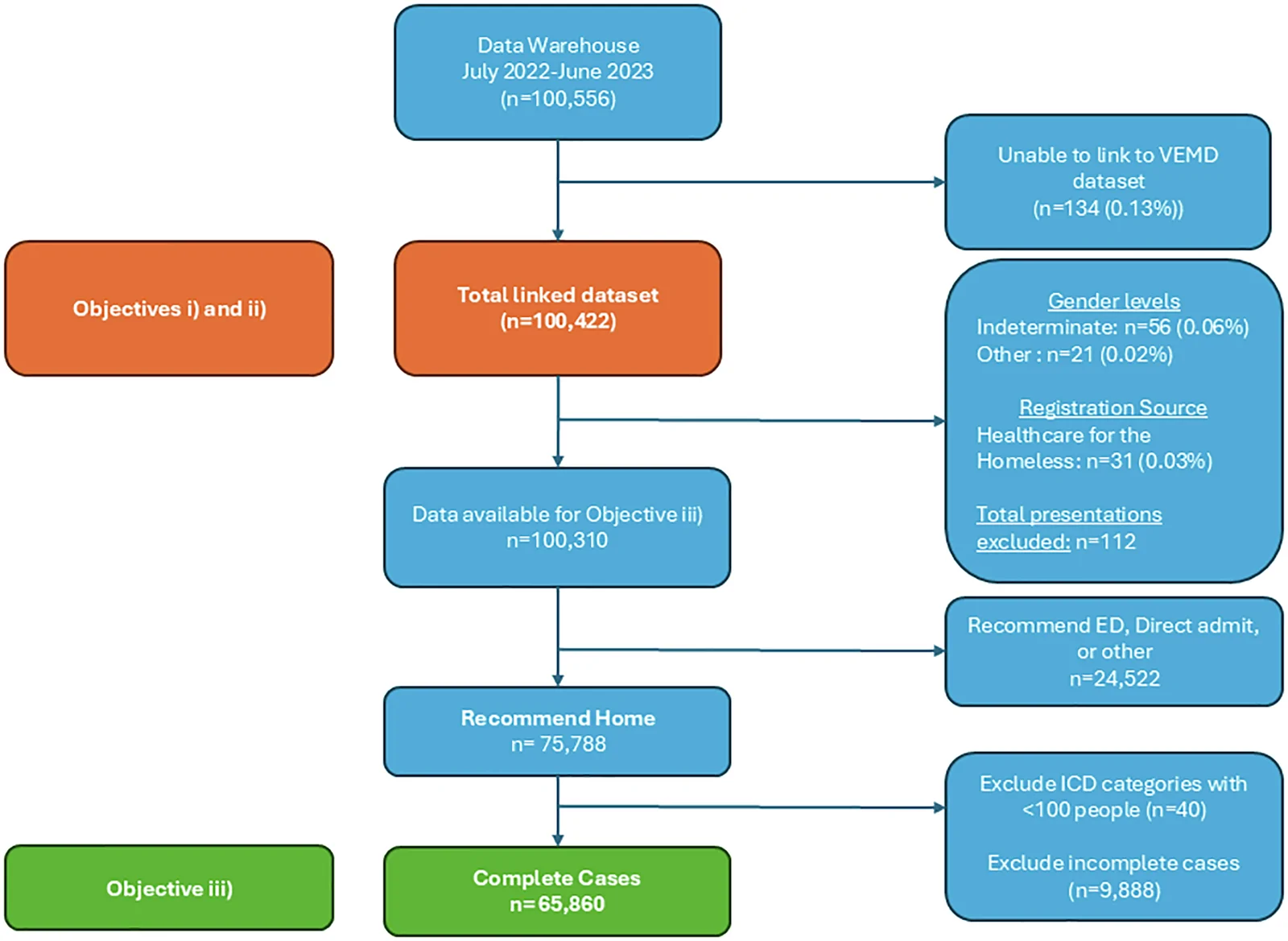
Data availability for each study objective. Flow diagram showing derivation of the analytical cohorts used for Objectives i–iii, including record linkage exclusions, and complete-case selection for analysis of factors associated with unplanned ED presentation within 48 h (objective iii).

### Sample characteristics

Table 1 presents sample characteristics. Most patients were self-referred (n = 60,656, 60%). The patients were predominantly female (n = 58,475, 58%), with 42% (n = 41,866) identifying as male and a small proportion categorised as indeterminate (n = 56, 0.05%) or other (n = 21, 0.02%). Median (interquartile range) age was 34 (55) years, ranging from 0 to 106 years. The largest age category being those aged 0–10 years (n = 26,275, 26%), followed by 31–40 years (n = 13,253, 13%). The smallest age group were those greater that 90 years (n = 4,672, 5%). The majority of patients were born in Australia (n = 87,805, 88%), and 97% (n = 93,099) described English as their first language, with 2% (n = 1703) requiring an interpreter. Socio-economic status varied across categories, with 47% being in the ‘low’ (n = 23,352) or ‘high’ (n = 23,173) quintiles. The most common ICD category included symptoms and abnormal clinical findings (n = 29,834,32%), codes for special purposes (n = 12,481, 14%) or respiratory diseases (n = 10,424,11%).

**Table 1:** Discharge destination across demographic and clinical characteristics for patients presenting to the VVED between 1 July 2022 and 23 June 2023.

Characteristic	N	Overall N = 100,422	Home N = 75,871 (76%)	ED N = 15,353 (15%)	Direct Admit N = 326 (0.3%)	Other N = 8872 (9%)
Gender	100,418					
Female		58,475 (100%)	44,541 (76%)	8708 (15%)	177 (0.3%)	5049 (8.6%)
Indeterminate		56 (100%)	44 (79%)	5 (8.9%)	0 (0%)	7 (13%)
Male		41,866 (100%)	31,268 (75%)	6638 (16%)	149 (0.4%)	3811 (9.1%)
Other		21 (100%)	14 (67%)	2 (9.5%)	0 (0%)	5 (24%)
(Missing)		4	4	0	0	0
Age (Mean (SD))	100,422	40.18 (30.22)	39.23 (29.99)	46.65 (31.31)	35.98 (31.16)	37.20 (28.74)
Age category	100,422					
0–10		26,275 (100%)	20,532 (78%)	3227 (12%)	110 (0.4%)	2406 (9.2%)
11–20		5068 (100%)	3962 (78%)	619 (12%)	17 (0.3%)	470 (9.3%)
21–30		10,347 (100%)	7813 (76%)	1361 (13%)	25 (0.2%)	1148 (11%)
31–40		13,253 (100%)	10,219 (77%)	1753 (13%)	38 (0.3%)	1243 (9.4%)
41–50		8804 (100%)	6762 (77%)	1242 (14%)	29 (0.3%)	771 (8.8%)
51–60		7374 (100%)	5505 (75%)	1184 (16%)	23 (0.3%)	662 (9.0%)
61–70		6925 (100%)	5138 (74%)	1187 (17%)	19 (0.3%)	581 (8.4%)
71–80		8432 (100%)	6004 (71%)	1719 (20%)	23 (0.3%)	686 (8.1%)
81–90		9272 (100%)	6508 (70%)	2082 (22%)	31 (0.3%)	651 (7.0%)
91+		4672 (100%)	3428 (73%)	979 (21%)	11 (0.2%)	254 (5.4%)
Birth Country	99,692					
Australia (inc External Territories)		87,805 (100%)	66,524 (76%)	13,340 (15%)	289 (0.3%)	7652 (8.7%)
New Zealand		641 (100%)	472 (74%)	106 (17%)	3 (0.5%)	60 (9.4%)
Other		11,246 (100%)	8340 (74%)	1771 (16%)	34 (0.3%)	1101 (9.8%)
(Missing)		730	535	136	0	59
English speaking	96,075					
N		2976 (100%)	2121 (71%)	575 (19%)	13 (0.4%)	267 (9.0%)
Y		93,099 (100%)	70,527 (76%)	14,102 (15%)	306 (0.3%)	8164 (8.8%)
(Missing)		4347	3223	676	7	441
Interpreter requested	100,422					
N		98,719 (100%)	74,683 (76%)	15,015 (15%)	321 (0.3%)	8700 (8.8%)
Y		1703 (100%)	1188 (70%)	338 (20%)	5 (0.3%)	172 (10%)
Marital status	70,966					
Unpartnered		48,641 (100%)	36,877 (76%)	6697 (14%)	184 (0.4%)	4883 (10%)
Partnered		22,325 (100%)	17,054 (76%)	3189 (14%)	68 (0.3%)	2014 (9.0%)
(Missing)		29,456	21,940	5467	74	1975
Socio-economic status	98,575					
Very High		18,383 (100%)	14,226 (77%)	2368 (13%)	40 (0.2%)	1749 (9.5%)
High		23,173 (100%)	17,429 (75%)	3571 (15%)	70 (0.3%)	2103 (9.1%)
Medium		17,648 (100%)	13,220 (75%)	2752 (16%)	41 (0.2%)	1635 (9.3%)
Low		23,352 (100%)	17,778 (76%)	3540 (15%)	99 (0.4%)	1935 (8.3%)
Very low		16,019 (100%)	11,820 (74%)	2818 (18%)	66 (0.4%)	1315 (8.2%)
(Missing)		1847	1398	304	10	135
Regional category	98,093					
Metro		74,100 (100%)	56,377 (76%)	10,862 (15%)	180 (0.2%)	6681 (9.0%)
Regional		7021 (100%)	5225 (74%)	1115 (16%)	36 (0.5%)	645 (9.2%)
Rural/Remote		16,972 (100%)	12,497 (74%)	3037 (18%)	101 (0.6%)	1337 (7.9%)
(Missing)		2329	1772	339	9	209
Registration source	100,422					
Self-referral/self-registration		60,656 (100%)	46,378 (76%)	7499 (12%)	196 (0.3%)	6583 (11%)
Healthcare Professional		39,766 (100%)	29,493 (74%)	7854 (20%)	130 (0.3%)	2289 (6%)
ICD-10-AM chapter grouping	91,874					
Symptoms, signs and abnormal clinical and laboratory findings, not elsewhere classified		29,834 (100%)	22,505 (75%)	7124 (24%)	104 (0.3%)	101 (0.3%)
Certain conditions originating in the perinatal period		139 (100%)	102 (73%)	34 (24%)	3 (2.2%)	0 (0%)
Certain infectious and parasitic diseases		6932 (100%)	6451 (93%)	458 (6.6%)	21 (0.3%)	2 (<0.1%)
Codes for special purposes		12,481 (100%)	11,211 (90%)	1077 (8.6%)	23 (0.2%)	170 (1.4%)
Congenital malformations, deformations and chromosomal abnormalities		8 (100%)	5 (63%)	3 (38%)	0 (0%)	0 (0%)
Diseases of the blood and blood-forming organs and certain disorders involving the immune mechanism		193 (100%)	133 (69%)	59 (31%)	1 (0.5%)	0 (0%)
Diseases of the circulatory system		2473 (100%)	1689 (68%)	764 (31%)	15 (0.6%)	5 (0.2%)
Diseases of the digestive system		3493 (100%)	2900 (83%)	570 (16%)	14 (0.4%)	9 (0.3%)
Diseases of the ear and mastoid process		1268 (100%)	1210 (95%)	54 (4.3%)	2 (0.2%)	2 (0.2%)
Diseases of the eye and adnexa		824 (100%)	676 (82%)	143 (17%)	5 (0.6%)	0 (0%)
Diseases of the genitourinary system		2665 (100%)	2162 (81%)	483 (18%)	17 (0.6%)	3 (0.1%)
Diseases of the musculoskeletal system and connective tissue		3027 (100%)	2510 (83%)	501 (17%)	15 (0.5%)	1 (<0.1%)
Diseases of the nervous system		1379 (100%)	1033 (75%)	334 (24%)	8 (0.6%)	4 (0.3%)
Diseases of the respiratory system		10,424 (100%)	9226 (89%)	1148 (11%)	42 (0.4%)	8 (<0.1%)
Diseases of the skin and subcutaneous tissue		3315 (100%)	2951 (89%)	350 (11%)	9 (0.3%)	5 (0.2%)
Endocrine, nutritional and metabolic diseases		571 (100%)	352 (62%)	216 (38%)	2 (0.4%)	1 (0.2%)
Factors influencing health status and contact with health services		2497 (100%)	2412 (97%)	75 (3.0%)	1 (<0.1%)	9 (0.4%)
Injury, poisoning and certain other consequences of external causes		8149 (100%)	6489 (80%)	1615 (20%)	36 (0.4%)	9 (0.1%)
Mental and behavioural disorders		1529 (100%)	1291 (84%)	231 (15%)	4 (0.3%)	3 (0.2%)
Neoplasms		52 (100%)	41 (79%)	10 (19%)	1 (1.9%)	0 (0%)
Pregnancy, childbirth and the puerperium		621 (100%)	513 (83%)	104 (17%)	3 (0.5%)	1 (0.2%)
(Missing)		8548	9	0	0	8539

### Discharge destination

Table 1 presents the breakdown of discharge destination across demographic and clinical characteristics. The majority of patients (76%, n = 75,871) were advised to remain at home, while 15% (n = 15,353) were advised to present to the ED. A smaller proportion were referred for direct hospital admission (0.3%, n = 326), and 9% (n = 8872) were classified under other categories.

### Downstream ED utilisation following a VVED presentation overall hospital avoidance

Table 2 and Fig. 2 summarise hospital utilisation within 48 h and 7 days. Within 48 h of their VVED presentation, 18,582 patients (18.5%) presented to a physical ED, representing an overall avoidance rate of 81.5%. There were 9639 (9.6%) hospital admissions recorded within 48 h. The majority of ED presentations within this timeframe were among patients who had been advised to attend the ED (n = 10,306; 67.1% of all ED presentations), and these individuals also accounted for the majority of hospital admissions (n = 5539; 36.1% of all admissions) within 48 h.

**Table 2:** Discharge outcomes and hospital presentations for patients presenting to the VVED between 1 July 2022 and 23 June 2023.

Discharge disposition	Registration pathway	Total	48 Hours		7 Days	
			ED n (%)	Adm n (%)	ED n (%)	Adm n (%)
Home	Self-referral	46,378	3655 (7.9%)	1399 (3%)	5100 (11%)	2330 (5%)
	Ambulance	21,364	2028 (9.5%)	1351 (6.3%)	2816 (13.2%)	2066 (9.7%)
	HCP	834	59 (7.1%)	20 (2.4%)	90 (10.8%)	46 (5.5%)
	Redirected 000	2044	105 (5.1%)	76 (3.7%)	137 (6.7%)	101 (4.9%)
	Healthcare for the homeless	21	0 (0%)	0 (0%)	0 (0%)	0 (0%)
	RACF	2302	184 (8%)	159 (6.9%)	271 (11.8%)	247 (10.7%)
	Urgent Care Centre	2928	171 (5.8%)	142 (4.8%)	253 (8.6%)	219 (7.5%)
	**Total**	**75,871**	**6202 (8.2%)**	**3147 (4.1%)**	**8667 (11.4%)**	**5009 (6.6%)**
Direct Admit	Self-referral	196	70 (35.7%)	25 (12.8%)	78 (39.8%)	28 (14.3%)
	Ambulance	54	25 (46.3%)	17 (31.5%)	27 (50%)	21 (38.9%)
	HCP	25	4 (16%)	3 (12%)	4 (16%)	3 (12%)
	Redirected 000	3	1 (33.3%)	0 (0%)	1 (33.3%)	0 (0%)
	Healthcare for the homeless	1	0 (0%)	0 (0%)	0 (0%)	0 (0%)
	RACF	4	2 (50%)	1 (25%)	2 (50%)	1 (25%)
	Urgent Care Centre	43	7 (16.3%)	18 (41.9%)	8 (18.6%)	18 (41.9%)
	**Total**	**326**	**109 (33.4%)**	**64 (19.6%)**	**120 (36.8%)**	**71 (21.8%)**
ED	Self-referral	7499	5593 (74.6%)	2555 (34.1%)	5706 (76.1%)	2754 (36.7%)
	Ambulance	5758	3404 (59.1%)	2128 (37%)	3434 (59.6%)	2228 (38.7%)
	HCP	144	89 (61.8%)	58 (40.3%)	90 (62.5%)	60 (41.7%)
	Redirected 000	409	158 (38.6%)	91 (22.2%)	164 (40.1%)	102 (24.9%)
	Healthcare for the homeless	8	7 (87.5%)	4 (50%)	7 (87.5%)	4 (50%)
	RACF	628	423 (67.4%)	323 (51.4%)	424 (67.5%)	331 (52.7%)
	Urgent Care Centre	907	632 (69.7%)	380 (41.9%)	640 (70.6%)	397 (43.8%)
	**Total**	**15,353**	**10,306 (67.1%)**	**5539 (36.1%)**	**10,465 (68.2%)**	**5876 (38.3%)**
Other	Self-referral/self-registration	6583	1355 (20.6%)	533 (8.1%)	1549 (23.5%)	689 (10.5%)
	Ambulance referral	1637	501 (30.6%)	296 (18.1%)	544 (33.2%)	332 (20.3%)
	HCP referral	85	16 (18.8%)	8 (9.4%)	16 (18.8%)	9 (10.6%)
	Redirected 000	277	25 (9%)	15 (5.4%)	29 (10.5%)	20 (7.2%)
	Healthcare for the homeless	1	0 (0%)	0 (0%)	0 (0%)	0 (0%)
	RACF referral	157	37 (23.6%)	23 (14.6%)	40 (25.5%)	26 (16.6%)
	Urgent care centre referral	132	31 (23.5%)	14 (10.6%)	35 (26.5%)	19 (14.4%)
	**Total**	**8872**	**1965 (22.1%)**	**889 (10%)**	**2213 (24.9%)**	**1095 (12.3%)**

**Fig. 2:**
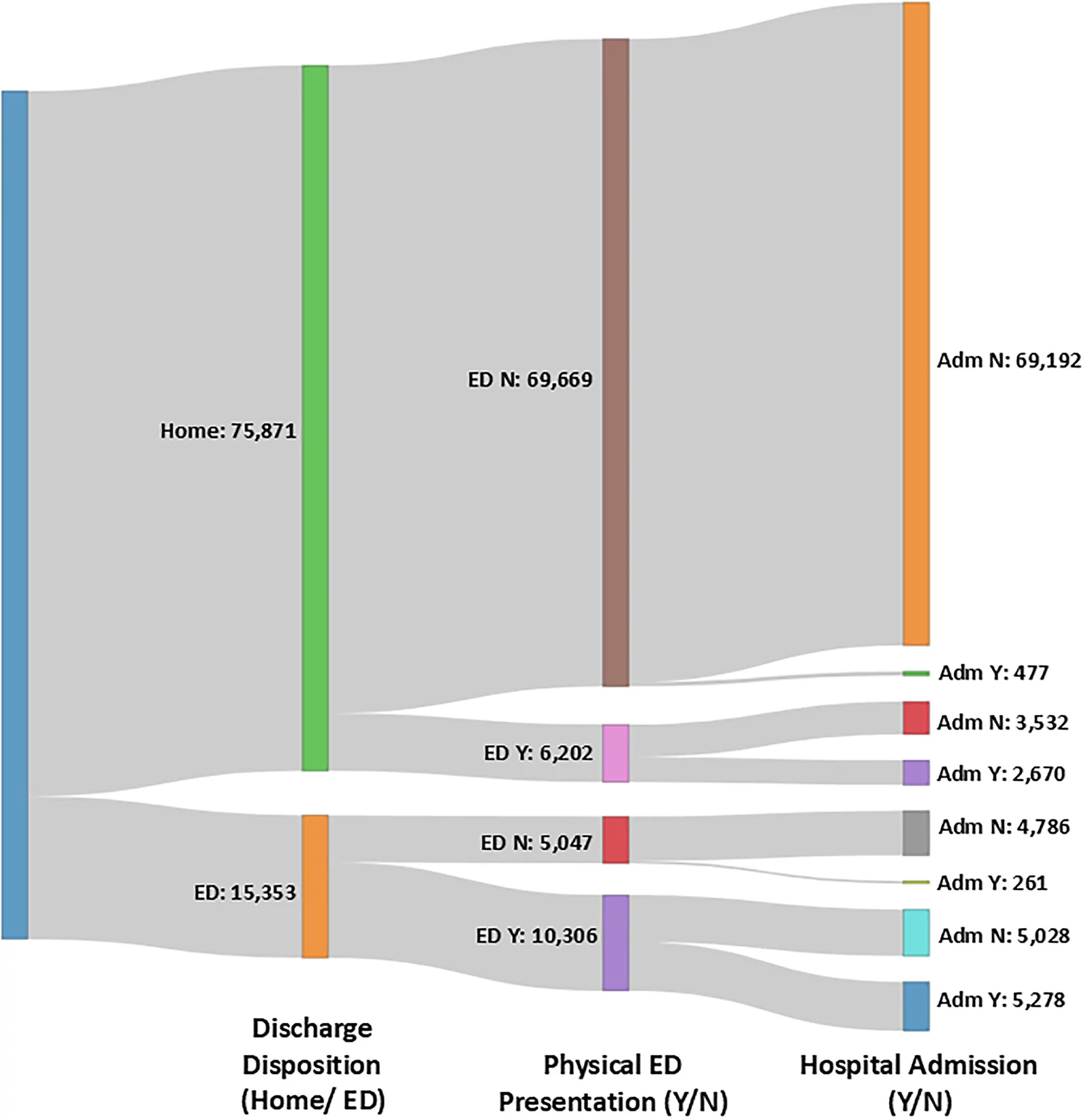
Alluvial diagram illustrating hospital utilisation (48 h) of VVED consultations where discharge disposition was defined as Home (Care in place) or ED. Adm = Hospital admission; ED: Emergency Department.

### Planned and unplanned hospital utilisation (48 h, 7 days)

#### Unplanned ED attendance and hospitalisation

Among patients discharged home (n = 75,871), 8.2% (n = 6202) re-presented to the ED and 4.1% (n = 3147) were admitted to hospital within 48 h, increasing to 11.4% (n = 8667) and 6.6% (n = 5009) respectively within 7 days. Ambulance referrals had the highest rates of both ED re-presentation (9.5% [n = 2028] at 48 h; 13.2% [n = 2816] at 7 days) and admissions (6.3% [n = 1351] and 9.7% [n = 2066]), followed by referrals from residential aged care facilities (8.0% [n = 184] and 11.8% [n = 271] ED re-presentation; 6.9% [n = 159] and 10.7% [n = 247] admissions). Self-referrals comprised the largest group (n = 46,378), with 7.9% (n = 3655) attending the ED and 3.0% (n = 1399) being admitted within 48 h, rising to 11.0% (n = 5100) and 5.0% (n = 2330) within 7 days.

#### Planned ED attendance

Among patients with a discharge destination of ED (n = 15,353), 67.1% (n = 10,306) attended the ED within 48 h, and 36.1% (n = 5539) were admitted to hospital, increasing slightly to 68.2% (n = 10,465) and 38.3% (n = 5876) within 7 days. Attendance within 48 h was highest among those referred from healthcare for the homeless services (87.5% [n = 7]) and self-referrals (74.6% [n = 5593]), while admission rates were highest for referrals from residential aged care facilities (51.4% [n = 323] at 48 h; 52.7% [n = 331] at 7 days) and healthcare for the homeless (50% at both time points [n = 4]). Redirected emergency calls had the lowest ED attendance (38.6% [n = 158]) and admission (22.2% [n = 91]) rates at 48 h.

### Factors associated with unplanned ED presentations within 48 h among patients initially managed in the community following a VVED presentation

Fig. 1 summarises the derivation of the cohort used to examine factors associated with unplanned ED presentation within 48 h of a VVED consultation. Of the 100,422 linked VVED presentations, 75,788 patients were discharged home and therefore eligible for analysis of unplanned ED utilisation. After excluding presentations with incomplete data (n = 9888) and diagnostic categories with fewer than 100 observations (n = 40), the final complete-case cohort comprised 65,860 presentations, of which 5688 (8.6%) resulted in an unplanned ED presentation within 48 h (Supplementary Table S2).

Primary diagnosis category was the only variable associated with unplanned ED presentation within 48 h in univariable analyses (Supplementary Table S2), and remained the only variable associated with unplanned ED presentation in the multivariable model after adjustment for English-speaking status, registration source, and regionality.

Compared with patients presenting with symptoms, signs and abnormal clinical and laboratory findings not elsewhere classified (R00–R99), lower odds of unplanned ED presentation were observed among patients with factors influencing health status and contact with health services (Z00–Z99; OR 0.21, 95% CI 0.11–0.40), diseases of the skin and subcutaneous tissue (L00–L99; OR 0.27, 95% CI 0.16–0.44), diseases of the ear and mastoid process (H60–H99; OR 0.30, 95% CI 0.14–0.64), codes for special purposes (U04–U49, U78–U88, U91–U92; OR 0.32, 95% CI 0.23–0.45), diseases of the circulatory system (I00–I99; OR 0.44, 95% CI 0.24–0.80), diseases of the respiratory system (J00–J99; OR 0.61, 95% CI 0.46–0.79), and injury, poisoning and certain other consequences of external causes (S00–T98; OR 0.61, 95% CI 0.45–0.84) (Fig. 3).

**Fig. 3:**
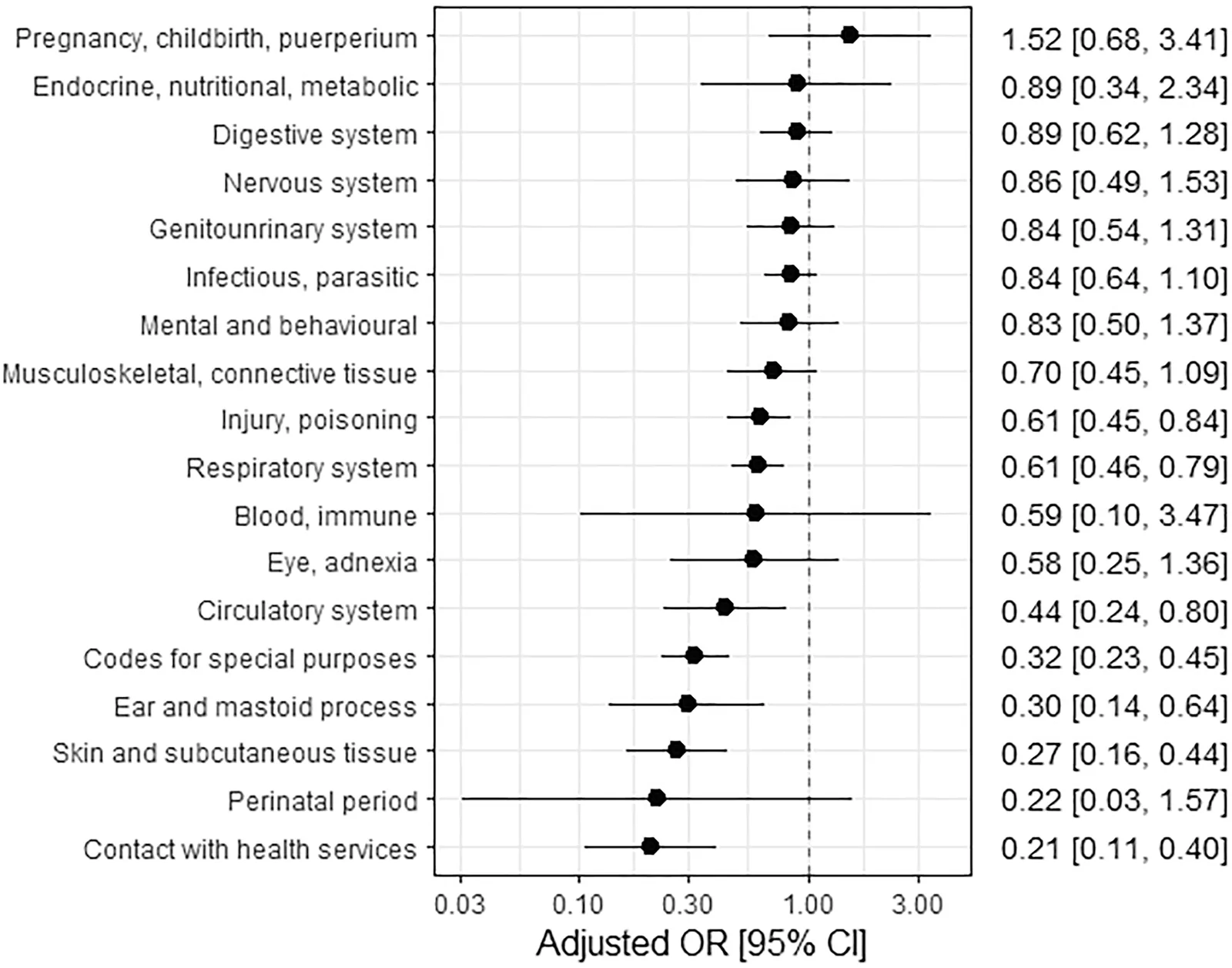
Forest plot illustrating multivariable logistic regression results for the association between ICD-Chapter groupings and hospital ED presentations within 48 h of VVED presentation, for those initially managed in the community (n = 65,860). Referent diagnosis is “symptoms, signs and abnormal clinical and laboratory findings not elsewhere classified (R00–R99)”. Data presented as odds ratios (OR) with 95% confidence intervals (CI).

## Discussion

This study provides real-world evidence on short-term hospital utilisation following consultations with the VVED. Across a large cohort of over 100,000 VVED records, more than 8/10 (81.5%) avoided a hospital presentation within 48 h, regardless of their discharge disposition. The majority of patients (76%) did not require escalation to physical emergency care, and of these, only 8.2% and 11.4% presented to a physical ED within 48 h and 7 days respectively. Primary ICD-Chapter grouping was the only factor associated with 48 h physical ED re-presentations for those managed in the community.

Overall, 81.5% of patients did not attend a physical ED within 48 h of their index VVED consultation (ED avoidance rate). This finding indicates that more than four in five patients who accessed the VVED did not subsequently present to a hospital ED in the short term, regardless of their initial discharge disposition. At a population scale of over 100,000 presentations, this may represent a meaningful reduction in ED attendances. This pattern aligns with statewide trends demonstrating a reduction in lower-acuity ED presentations alongside increasing acuity among patients presenting to hospital EDs in Victoria.^27^ Recent analysis of more than 11 million ED presentations in Victoria from 2018 to 2024 found declining triage category 4–5 attendances (low acuity), with avoidance strategies such as the VVED cited as a likely contributor.^27^ Notably, Victoria was the only state to record a fall in ambulance incidents in 2024–25. The Australian Medical Association (AMA) and Ambulance Victoria attributed this 5.4 reduction in part to increased active management of lower-acuity cases, with the VVED representing one of Ambulance Victoria's largest alternative care pathways.^28^^,^^29^ Taken together, these findings suggest that ED avoidance through the VVED was not driven by under-triage, but by appropriate clinical selection and management, as reflected by the relatively low rates of unplanned ED presentation among patients advised to remain at home.

The observed unplanned ED presentation rate of 8.2% amongst patients advised to remain at home is comparable to, and in some cases lower than, rates reported following discharge from physical ED care in Australia and internationally. Reported re-presentation rates following ED discharge commonly range from approximately 5% in Australian settings to over 30% internationally.^16^^,^^30^ Additionally, when considering remote-based triage models, our rates are comparable to those reported for Australian nurse-led telephone triage services, where approximately 7% of patients advised to self-care attended an ED or hospital within 24 h.^31^ Our rates were also lower than the 10.5% unplanned ED presentation rate reported for patients triaged to self-care or primary care pathways through a nurse-only virtual urgent care model in Britain^32^ and the 13.7% at 72 h (16.5% at 7 days) found by a large urgent care pilot in Canada.^13^ These findings indicate that clinician-led, video-based virtual emergency care can safely manage selected low-acuity presentations at home, achieving unplanned ED presentation rates within the range observed following discharge from physical emergency departments and other remotely delivered models of care.

In both univariable and multivariable analyses, ICD-10 diagnostic category was the only factor independently associated with re-presentation to a physical ED within 48 h, consistent with findings from the in-person emergency setting.^33^

Conditions amenable to diagnosis through history and visual inspection, including skin disorders and minor injuries, showed the greatest reductions in re-presentation, supporting their particular suitability for videohealth. Conditions such as suspected otitis media, where acute symptomatic management can be initiated virtually and patients streamed to community-based care, similarly demonstrated significantly lower re-presentation odds.

The only diagnostic category with an odds ratio above 1.0 was pregnancy, childbirth and puerperium, though this did not reach statistical significance and the wide confidence interval likely reflects limited case numbers. Early pregnancy complications, particularly bleeding in early pregnancy, present a distinct challenge where management depends on timely access to ultrasound and early pregnancy unit or GP care; when these pathways are unavailable or delayed, re-presentation to a physical ED may be the patients only option.

Patients coded under non-specific diagnostic categories (R00–R99) may represent those whose illness had not yet adequately declared itself at the time of VVED presentation. These patients are routinely advised to re-present should symptoms evolve, and their higher relative re-presentation rate likely reflects appropriate safety-netting by the VVED rather than a failure of virtual care, with subsequent ED attendance representing the intended clinical pathway as illness trajectory becomes clearer.

Unplanned hospital admission following VVED consultation was more common among patients referred via healthcare professional pathways compared with those who self-referred. These registrations include those initiated by paramedics, RACFs, urgent care centres and other community-based clinicians. Referrals initiated by paramedics and RACFs are well recognised to involve older, frailer patients with multimorbidity and a higher baseline risk of hospital admission compared with community self-referrals.^34^, ^35^, ^36^, ^37^ In many cases, transfer to emergency care is prompted by acute clinical deterioration that cannot be safely managed within the patient's usual residence (e.g., RACF),^38^ consistent with established escalation directives from VVED clinicians, that advise urgent hospital assessment if a resident’s condition worsens. When deterioration occurs, these patients are more likely to present with significant physiological instability or complex care requirements, making hospital admission an appropriate and expected outcome rather than an avoidable escalation. While these patients demonstrated higher rates of hospital admission, the majority of this cohort (74%) were discharged home without requiring an ED attendance or admission within 48 h. This suggests that the VVED was able to safely manage a substantial proportion of these clinically complex patients, without requiring escalation to physical ED care.

Interestingly, linked data indicated that 33% of patients did not attend an ED within 48 h despite being advised to do so. This level of non-compliance is remarkably consistent with findings from Australian telephone triage services, where 34% of callers advised to “attend ED immediately” did not comply to advice within 24 h.^39^ While the majority of patients adhered to clinical advice, this high proportion suggests that decision-making following a virtual consultation is influenced by a range of factors that may go beyond clinician recommendation. Potential contributors include the patients’ perceptions of illness severity, trust in medical advice, communication quality, barriers to accessing in-person care (such as geographical distance or regionality, transport limitations, or ambulance costs), patient re-evaluation of symptom severity, preference for community-based alternatives (e.g., general practice or urgent care centres), or symptom improvement following the consultation.^31^^,^^40^^,^^41^ It is important to note that private hospital ED presentations are not captured in the linked datasets, which may also partly account for this finding. These findings highlight the need to understand patient decision-making following virtual consultations and suggest there might be opportunities for strengthening communication, building trust,^42^ and enhancing support mechanisms for patients triaged to in-person care. Improving these may help enable clinical recommendations to be more readily acted upon and reduce the potential risks from non-attendance.

Further investigation is warranted to examine the clinical outcomes of patients who did not attend the ED despite being advised to do so, as this group may provide important insights into both the safety and the limitations of virtual emergency models.^31^ While VVED achieved ED re-presentation rates comparable to or better than other physical or remote models, additional safety indicators, including mortality, are required. Current evidence on VVED’s impact on physical ED presentations is largely based on theoretical extrapolation rather than empirical observation.^27^ Although establishing causality is challenging, analyses such as statewide time-series evaluations^7^ of physical ED presentations could strengthen the evidence base and provide a clearer temporal link to VVED implementation, a critical step in understanding causation.^43^ Further work is needed to assess the health economic impact of a statewide VVED model. Existing evidence is limited; a prior, pilot modelled evaluation suggested potential cost neutrality or savings^44^; however, this analysis was based on a small cohort of 188 patients over approximately four months and relied on conservative assumptions regarding hospital admissions. Larger evaluations using linked, population-level data that directly measure hospital admissions, admission type, and longer-term hospital utilisation are therefore required to more accurately quantify system-level cost impacts and return on investment at scale. Qualitative exploration of clinician and patient experience would provide insight into service improvement opportunities not captured by administrative data, including the drivers of differential utilisation and outcomes across subpopulations. Finally, because the time-windows for our multivariable modelling was set to align with established benchmarks,^15^ logistic regression was used to model the occurrence of events rather than their timing. Further work could be conducted to assess whether the time to ED presentation was associated with demographic or clinical factors.

The main strength of this study is that it represents a large, diverse real-world cohort of over 100,000 VVED presentations across an entire state. This study used routinely linked administrative data generated through a routine record linkage system. While the use of linked data addresses key methodological limitations of studies unable to capture patient re-presentations to other health services^16^ (potentially contributing to the large variation in reported rates of re-presentation) residual record linkage error may remain, including missed and false matches due to incomplete or inaccurate identifiers. Linkage error may be non-random across population subgroups and could introduce misclassification or selection bias. In addition, routinely collected administrative data may be subject to coding inaccuracies or missing contextual variables, such as patient preferences or social barriers.

The findings may also not be generalisable beyond the Australian context, particularly given variations in ED access models, ambulance funding structures, variable technology access and telehealth maturity internationally. In addition, these outcomes were based on hospital utilisation (ED presentations and admissions) and do not capture clinical outcomes, patient safety, or quality-of-care measures. Presentations to private emergency departments and related admissions are not captured in the VEMD or VAED datasets. However, private emergency department use accounts for only a small proportion of total emergency presentations in Australia.^45^

The population was predominantly Australian-born and English-speaking, therefore underrepresenting culturally and linguistically diverse populations compared with the broader Victorian population, where approximately 30% of residents are overseas-born and 27% speak a language other than English at home.^46^ Patients presenting to VVED were predominantly female (58%), consistent with established patterns of higher healthcare utilisation among women in both ED and broader healthcare settings in Australia and internationally.^47^^,^^48^ Whether this reflects differential health-seeking behaviour, greater comfort with telehealth, or underlying differences in presentation acuity warrants further investigation. Patients from all socioeconomic quintiles were represented in the cohort. Previous work has demonstrated relatively even utilisation of the VVED across socioeconomic groups overall, although referral pathways differed, with patients from more disadvantaged areas more likely to access the service via healthcare provider referral than self-referral.^49^

Although this evaluation complied with applicable ethics approvals and data governance requirements for the use of routinely collected administrative health data, it did not include formal Aboriginal and Torres Strait Islander governance or Aboriginal investigators. Contemporary best practice recognises that research involving Aboriginal and Torres Strait Islander data, including administrative datasets, should incorporate Indigenous data governance principles, leadership, and stewardship throughout the research lifecycle. Future evaluations of virtual models of care should seek to embed Aboriginal and Torres Strait Islander governance to strengthen cultural integrity, support Indigenous data sovereignty, and ensure that research is undertaken in ways that are aligned with community priorities and deliver meaningful benefit.

This study provides a snapshot of downstream hospital utilisation during the first full year of statewide VVED operation. The findings should be interpreted within the context of this defined implementation phase. Finally, while 48-h and 7-day follow-up captures short-term utilisation, longer-term outcomes and reasons for non-adherence to clinical recommendations were not assessed.

### Conclusion

Overall, this large, real-world, linked data analysis demonstrates that most patients presenting to the VVED were managed in the community, with low rates of subsequent unplanned ED presentation and hospital admission. These findings support the VVED as a scalable complement to traditional emergency department services with potential to meaningfully reduce pressure on physical EDs at a population level. Ongoing evaluation is required to further understand patient decision-making, compliance to clinical advice, and longer-term clinical and safety outcomes, including mortality, particularly among higher-risk groups, to ensure virtual emergency care continues to deliver safe, equitable, and high-quality urgent care as it continues to grow.

## Contributors

LS, RJ, JB, DB and AS conceptualised this study. JB, DB and AS curated the data, MS and AS analysed and verified the data. LS, RJ, JB, JH, ZN and AS assessed the methods and interpreted the results. LS wrote the original manuscript. LS, RJ, JB, JH, ZN, AS, JL, SM, DB and MS contributed to the drafting and critical review of the manuscript. All authors approved the final manuscript for publication. LS, RJ, JB, DB and AS obtained funding for the study.

## Data sharing statement

Data used for this analysis will be made available upon reasonable request to the corresponding author.

## Declaration of interests

LMS, JH, JL, and SM are employed as salaried clinicians within the Victorian Virtual Emergency Department (VVED) at Northern Health, Melbourne, Australia, the service described and evaluated in this manuscript. None has a financial or commercial interest in the outcomes of this research.

LMS declares participation on the La Trobe University Digital Health Course Advisory Committee (2023–2025).

ZN is employed as Director of Research and Evaluation at Ambulance Victoria. ZN has no financial or commercial interest in the outcomes of this research.

MS and DB declare no conflicts of interest.

## Appendix A Supplementary data

Supplementary Tables S1–S3 and Equations 1 and 2
