# Supplementary material for: Outcomes of a state-wide virtual emergency department in Victoria, Australia: a linked data analysis of emergency department utilisation and hospital use
Source: Lancet Reg Health West Pac. 2026 Sep 19;74:101985. doi: 10.1016/j.lanwpc.2026.101985 (PMC13628662; doi:10.1016/j.lanwpc.2026.101985)
Supplement: Supplementary file 1 [file mmc1.pdf]

Supplementary Material

Supplementary Tables

Supplementary Table 1. TIDieR-Telehealth[23] description of the Victorian Virtual Emergency Department (VVED) model of care

TIDieR-Telehealth item	Description of the VVED
**1. Brief name**	Victorian Virtual Emergency Department (VVED)
**2. Why**	Developed to provide an alternative to in-person emergency care for non-life-threatening conditions, improve access to emergency care, alleviate emergency department overcrowding, and reduce unnecessary physical ED presentations. Initially developed in response to COVID-19 pressures before expanding to broader emergency care.
**3. What (materials)**	Patients or Healthcare Providers access the service via the VVED website using a personal device with a camera (mobile phone, tablet or computer). Registration is completed online. Interpreter services are available. Registration pathways are provided through dedicated web links for patients and healthcare providers. Specific videoconferencing software/platform: *Healthdirect* is used for all video consultations.
**4. What (procedures)**	Patients or Healthcare Providers register online and enter a virtual waiting room. Self-referred patients undergo nurse-led video triage before receiving self-care advice, referral to primary care/community services, or escalation to a clinician consultation. Healthcare provider referrals bypass nurse triage and proceed directly to clinician consultation. Clinicians determine disposition, including discharge home, referral to community services, referral to a physical ED, or direct hospital admission where appropriate.
**5. Who provided**	Emergency nurses, emergency physicians, general practitioners, nurse practitioners, paediatric emergency physicians and paediatricians. Dedicated specialist pathways developed for paediatric and geriatric patients. Training included structured orientation, co-consults, ongoing education and access to a searchable clinical knowledge database.
**6. How**	Synchronous clinician-led video consultations.
**7. Where**	Patients access the VVED remotely from home, work or community settings. Clinicians deliver care remotely through the VVED. Ambulance referrals may occur from patients' homes or residential aged care facilities.
**8. When and how much**	Statewide service operates 24 hours/day, 7 days/week.
**9. Tailoring**	Referral pathways tailored according to referral source (self-referral, ambulance, Residential Aged Care Facility (RACF), General Practitioner (GP), urgent care centre and other healthcare providers). Dedicated paediatric (VVED Kids) and geriatric (GEDI) clinician pathways developed for specific patient groups.
**10. Modifications**	Service evolved from a local hospital virtual ED (October 2020) to a statewide service (March 2022). Progressive expansion included Ambulance Victoria, RACF, urgent care centre and Nurse-on-Call referral pathways, extended operating hours, specialist waiting rooms and statewide rollout.
**11. Planned fidelity**	Governance framework included iterative piloting, evaluation, refinement, expansion and integration into routine practice. Clear clinical guidelines, governance structures and ongoing clinician education supported implementation.
**12. Actual fidelity**	Formal evaluation of clinical outcomes and effectiveness is ongoing

Supplementary Table 2: Factors associated with unplanned ED Presentations within 48 hours.

Variable		^48 hr re-presentation 5,688/65,860 (8.64%)	Univariable	Multivariable
Gender			p=0.936	
	Female	3,391 / 38,714 (8.8%)	Referent	
	Male	2,297 / 27,146 (8.5%)	1.010 [0.798, 1.278]; p=0.936	
Age (per ten year increase)		NA	1.012 [0.973, 1.052]; p=0.556	
Age Category			p=0.811	
	0-10	1,687 / 18,837 (9.0%)	Referent	
	11-20	293 / 3,447 (8.5%)	1.367 [0.844, 2.212]; p=0.204	
	21-30	631 / 6,917 (9.1%)	0.965 [0.63, 1.479]; p=0.871	
	31-40	773 / 9,081 (8.5%)	1.057 [0.726, 1.54]; p=0.771	
	41-50	457 / 5,962 (7.7%)	0.931 [0.584, 1.483]; p=0.763	
	51-60	356 / 4,664 (7.6%)	0.871 [0.52, 1.457]; p=0.598	
	61-70	301 / 4,253 (7.1%)	0.929 [0.554, 1.557]; p=0.779	
	71-80	432 / 4,878 (8.9%)	1.139 [0.719, 1.804]; p=0.579	
	81-90	528 / 5,170 (10%)	1.368 [0.893, 2.095]; p=0.15	
	91+	230 / 2,651 (8.7%)	1.090 [0.597, 1.989]; p=0.78	
	N	5,508 / 64,294 (8.6%)	Referent	
	Y	180 / 1,566 (11%)	1.162 [0.556, 2.427]; p=0.690	
Birth Country			p=0.837	
	Australia (inc External Territories)	4,944 / 57,402 (8.6%)	Referent	
	New Zealand	44 / 444 (9.9%)	1.409 [0.418, 4.750]; p=0.580	
	Other	700 / 8,014 (8.7%)	1.045 [0.736, 1.482]; p=0.807	
English Speaking			p=0.199	p=0.261
	N	231 / 2,060 (11%)	Referent	Referent
	Y	5,457 / 63,800 (8.6%)	0.685 [0.385, 1.22]; p=0.199	0.716 [0.4, 1.283]; p=0.261
Interpreter Requested			p=0.308	
	N	5,561 / 64,715 (8.6%)	Referent	
	Y	127 / 1,145 (11%)	1.480 [0.696, 3.147]; p=0.308	
Socio-economic Status			p=0.935	
	Very High	967 / 12,155 (8.0%)	Referent	
	High	1,365 / 15,445 (8.8%)	1.058 [0.737, 1.519]; p=0.760	
	Medium	1,087 / 11,571 (9.4%)	1.157 [0.791, 1.691]; p=0.452	

Low	1,409 / 16,615 (8.5%)	0.996 [0.693, 1.431]; p=0.981	
Very low	860 / 10,074 (8.5%)	1.052 [0.705, 1.571]; p=0.803	
Regionality		p=0.150	p=0.531
Metro	4,608 / 50,804 (9.1%)	Referent	Referent
Regional	410 / 4,666 (8.8%)	0.917 [0.579, 1.45]; p=0.710	0.992 [0.627, 1.571]; p=0.974
Rural/Remote	670 / 10,390 (6.4%)	0.707 [0.497, 1.003]; p=0.052	0.809 [0.559, 1.172]; p=0.262
Registration Source		p=0.087	p=0.188
VED - Self-Referral/Self-Registration	3,526 / 43,398 (8.1%)	Referent	Referent
Ambulance Referral	1,726 / 16,292 (11%)	1.271 [1.024, 1.577]; p=0.030	1.159 [0.93, 1.445]; p=0.189
HCP Referral	59 / 784 (7.5%)	0.505 [0.218, 1.171]; p=0.111	0.445 [0.188, 1.05]; p=0.065
Redirected 000	89 / 1,445 (6.2%)	1.160 [0.630, 2.135]; p=0.633	1.208 [0.656, 2.227]; p=0.544
VRACF - RACF Referral	152 / 1,874 (8.1%)	0.895 [0.508, 1.577]; p=0.702	0.782 [0.443, 1.38]; p=0.395
VUCC - Urgent Care Centre Referral	136 / 2,067 (6.6%)	0.735 [0.364, 1.484]; p=0.390	0.806 [0.383, 1.697]; p=0.570
ICD-10-AM Chapter Grouping		p<0.001	p<0.001
Symptoms, signs and abnormal clinical and laboratory findings, not elsewhere classified (R00-R99)	2,223 / 19,338 (11%)	Referent	Referent
Certain conditions originating in the perinatal period (P00-P99)	9 / 100 (9.0%)	0.234 [0.034, 1.623]; p=0.142	0.222 [0.031, 1.573]; p=0.132
Certain infectious and parasitic diseases (A00-B99)	539 / 5866 (9.2%)	0.823 [0.631, 1.073]; p=0.15	0.840 [0.643, 1.098]; p=0.203
Codes for special purposes (U04-U49, U78-U88, U91, U92)	424 / 8932 (4.7%)	0.323 [0.231, 0.453]; p=0	0.322 [0.229, 0.451]; p<0.001
#Congenital malformations, deformations and chromosomal abnormalities (Q00-Q99)	0 / 5 (0%)	#NA	#NA
Diseases of the blood and blood-forming organs and certain disorders involving the immune mechanism (D50-D89)	12 / 119 (10%)	0.548 [0.092, 3.281]; p=0.51	0.591 [0.101, 3.468]; p=0.56
Diseases of the circulatory system (I00-I99)	93 / 1,409 (6.6%)	0.439 [0.238, 0.81]; p=0.008	0.436 [0.237, 0.803]; p=0.008
Diseases of the digestive system (K00-K99)	296 / 2,584 (11%)	0.87 [0.605, 1.251]; p=0.453	0.893 [0.620, 1.285]; p=0.541
Diseases of the ear and mastoid process (H60-H99)	66 / 1,067 (6.2%)	0.292 [0.135, 0.631]; p=0.002	0.296 [0.137, 0.642]; p=0.002
Diseases of the eye and adnexa (H00-H59)	43 / 623 (6.9%)	0.561 [0.242, 1.299]; p=0.177	0.583 [0.251, 1.356]; p=0.210
Diseases of the genitourinary system (N00-N99)	208 / 1,966 (11%)	0.811 [0.521, 1.261]; p=0.352	0.842 [0.540, 1.315]; p=0.450
Diseases of the musculoskeletal system and connective tissue (M00-M99)	194 / 2,157 (9.0%)	0.688 [0.441, 1.074]; p=0.100	0.701 [0.448, 1.095]; p=0.118
Diseases of the nervous system (G00-G99)	83 / 919 (9.0%)	0.852 [0.48, 1.512]; p=0.585	0.864 [0.487, 1.533]; p=0.618
Diseases of the respiratory system (J00-J99)	608 / 8,176 (7.4%)	0.596 [0.457, 0.777]; p<0.001	0.605 [0.464, 0.790]; p<0.001
Diseases of the skin and subcutaneous tissue (L00-L99)	179 / 2,698 (6.6%)	0.258 [0.157, 0.424]; p<0.001	0.267 [0.162, 0.441]; p<0.001
Endocrine, nutritional and metabolic diseases (E00-E89)	25 / 303 (8.3%)	0.886 [0.34, 2.311]; p=0.805	0.894 [0.342, 2.338]; p=0.820
Factors influencing health status and contact with health services (Z00-Z99)	68 / 2,209 (3.1%)	0.202 [0.104, 0.391]; p<0.001	0.206 [0.106, 0.401]; p<0.001
Injury, poisoning and certain other consequences of external causes (S00-T98)	467 / 5,810 (8.0%)	0.599 [0.439, 0.818]; p<0.001	0.614 [0.448, 0.841]; p=0.002
Mental and behavioural disorders (F00-F99)	94 / 1,104 (8.5%)	0.848 [0.512, 1.402]; p=0.519	0.831 [0.503, 1.374]; p=0.470
#Neoplasms (C00-D49)	3 / 35 (8.6%)	#NA	#NA
Pregnancy, childbirth and the puerperium (O00-O99)	57 / 480 (12%)	1.482 [0.663, 3.311]; p=0.337	1.518 [0.676, 3.409]; p=0.311

^Complete cases

#Removed from analysis due to low counts (n<100)

Supplementary Table 3: The RECORD statement – checklist of items, extended from the STROBE statement, that should be reported in observational studies using routinely collected health data.

	Item No.	STROBE items	Location in manuscript where items are reported	RECORD items	Location in manuscript where items are reported
**Title and abstract**					
	1	(a) Indicate the study's design with a commonly used term in the title or the abstract (b) Provide in the abstract an informative and balanced summary of what was done and what was found	Title and Abstract	RECORD 1.1: The type of data used should be specified in the title or abstract. When possible, the name of the databases used should be included.<br><br>RECORD 1.2: If applicable, the geographic region and timeframe within which the study took place should be reported in the title or abstract.<br><br>RECORD 1.3: If linkage between databases was conducted for the study, this should be clearly stated in the title or abstract.	Title: 'A linked data analysis'; Abstract: 'retrospective cohort study'<br><br>Abstract (methods)<br><br>Abstract: 'linked statewide administrative data'
**Introduction**					
Background rationale	2	Explain the scientific background and rationale for the investigation being reported	Introduction		Introduction, paragraphs 1–4, pp. 4-5
Objectives	3	State specific objectives, including any prespecified hypotheses	Introduction		Introduction, final paragraph (three dot-pointed objectives), p. 5
**Methods**					
Study Design	4	Present key elements of study design early in the paper	Methods		Methods- Study Design, p. 5
Setting	5	Describe the setting, locations, and relevant dates, including periods of recruitment, exposure, follow-up, and data collection	Methods		Methods- p. 5; Supplementary Table 1 (TIDieR-Telehealth)
Participants	6	*(a) Cohort study* - Give the eligibility criteria, and the sources and methods of selection of participants. Describe methods of follow-up<br>*Case-control study* - Give the eligibility criteria, and the sources and methods of case ascertainment and control selection. Give the rationale for the choice of cases and controls<br>*Cross-sectional study* - Give the eligibility criteria, and the sources and methods of selection of participants<br><br>*(b) Cohort study* - For matched studies, give matching criteria and number of exposed and unexposed<br>*Case-control study* - For matched studies, give	Methods<br><br>N/A	RECORD 6.1: The methods of study population selection (such as codes or algorithms used to identify subjects) should be listed in detail. If this is not possible, an explanation should be provided.<br><br>RECORD 6.2: Any validation studies of the codes or algorithms used to select the population should be referenced. If validation was conducted for this study and not published elsewhere, detailed methods and results should be provided.<br><br>RECORD 6.3: If the study involved linkage of databases, consider use of a flow diagram or other graphical display to demonstrate the data linkage process, including	Methods – Participants, p. 6<br><br>N/A<br><br>Methods – Data sources, access and linkage, p. 6; 99.87% linkage rate reported in Results

		matching criteria and the number of controls per case		the number of individuals with linked data at each stage.	
Variables	7	Clearly define all outcomes, exposures, predictors, potential confounders, and effect modifiers. Give diagnostic criteria, if applicable.	Methods	RECORD 7.1: A complete list of codes and algorithms used to classify exposures, outcomes, confounders, and effect modifiers should be provided. If these cannot be reported, an explanation should be provided.	Methods – Outcomes and Variables sections, pp. 5-6
Data sources/ measurement	8	For each variable of interest, give sources of data and details of methods of assessment (measurement).<br>Describe comparability of assessment methods if there is more than one group	Methods (Data Sources, Access and Linkage; Variables)		VEMD and VAED are standardised, mandatory state-wide datasets. Limitations section acknowledges potential coding inaccuracies and residual linkage error.
Bias	9	Describe any efforts to address potential sources of bias	Methods (Data Sources, Access and Linkage; Statistical Analysis) and Discussion (Strengths and Limitations).		Strengths/Limitations section, p. 21; linkage error, coding inaccuracy, complete-case analysis, missing data discussed
Study size	10	Explain how the study size was arrived at	Methods (Study Design; Participants); Results (first paragraph)		Study population determined by all VVED registrations during defined study period; n=100,422 after linkage exclusions (Figure 1)
Quantitative variables	11	Explain how quantitative variables were handled in the analyses. If applicable, describe which groupings were chosen, and why	Methods (Variables; Statistical Analysis)		Methods – Variables, p. 6-7; age presented continuously and categorised; SES in quintiles
Statistical methods	12	(a) Describe all statistical methods, including those used to control for confounding<br>(b) Describe any methods used to examine subgroups and interactions<br>(c) Explain how missing data were addressed<br>(d) *Cohort study* - If applicable, explain how loss to follow-up was addressed<br>*Case-control study* - If applicable, explain how matching of cases and controls was addressed<br>*Cross-sectional study* - If applicable, describe analytical methods taking account of sampling strategy<br>(e) Describe any sensitivity analyses	Methods (Statistical Analysis).<br><br>Methods (Statistical Analysis); Results (Tables 1 and 2)<br>Methods (Statistical Analysis); Results (Figure 1)<br>Methods (Participants<br><br>N/A		Methods – Data Analysis, pp. 7-8; mixed-effects logistic regression described
Data access and cleaning methods		..		RECORD 12.1: Authors should describe the extent to which the investigators had access to the database population used to create the study population.<br><br>RECORD 12.2: Authors should provide information on the data cleaning methods used in the study.	Methods p.6- data sources and access. Figure 1<br><br>Methods (Data Sources, Access and Linkage; Statistical Analysis); Results (Figure 1)
Linkage		..		RECORD 12.3: State whether the study included person-level, institutional-level, or other data linkage across two or more databases. The methods of	Methods p.6- linkage described; Results (first paragraph; Figure 1)

				linkage and methods of linkage quality evaluation should be provided.	
**Results**					
Participants	13	(a) Report the numbers of individuals at each stage of the study (*e.g.*, numbers potentially eligible, examined for eligibility, confirmed eligible, included in the study, completing follow-up, and analysed)<br>(b) Give reasons for non-participation at each stage.<br>(c) Consider use of a flow diagram	Results<br><br>Results<br><br>Results (figure 1)	RECORD 13.1: Describe in detail the selection of the persons included in the study (*i.e.,* study population selection) including filtering based on data quality, data availability and linkage. The selection of included persons can be described in the text and/or by means of the study flow diagram.	Results – first paragraph and Figure 1 (flow diagram), p. 8
Descriptive data	14	(a) Give characteristics of study participants (*e.g.*, demographic, clinical, social) and information on exposures and potential confounders<br>(b) Indicate the number of participants with missing data for each variable of interest<br>(c) *Cohort study* - summarise follow-up time (*e.g.*, average and total amount)	Results - Table 1<br><br>Results - Table 1<br><br>Methods (Outcomes; Participants)		Table 1 (sample characteristics with missing data indicated for each variable), p. 11
Outcome data	15	*Cohort study* - Report numbers of outcome events or summary measures over time<br>*Case-control study* - Report numbers in each exposure category, or summary measures of exposure<br>*Cross-sectional study* - Report numbers of outcome events or summary measures	Results		Results – Downstream ED utilisation, Table 2, pp. 14-15
Main results	16	(a) Give unadjusted estimates and, if applicable, confounder-adjusted estimates and their precision (e.g., 95% confidence interval). Make clear which confounders were adjusted for and why they were included<br>(b) Report category boundaries when continuous variables were categorized<br>(c) If relevant, consider translating estimates of relative risk into absolute risk for a meaningful time period	Results<br><br>Methods (variables)<br><br>Results (Downstream ED utilisation); Table 2		Figure 3 (forest plot); Supplementary Table 2 (univariable and multivariable ORs with 95% CIs)
Other analyses	17	Report other analyses done—e.g., analyses of subgroups and interactions, and sensitivity analyses	Results		Results – stratified by registration source and referral pathway throughout Tables 1 and 2
**Discussion**					
Key results	18	Summarise key results with reference to study objectives	Discussion, first paragraph		Discussion, first paragraph, p. 18
Limitations	19	Discuss limitations of the study, taking into account sources of potential bias or imprecision. Discuss both direction	Discussion (Strengths and Limitations)	RECORD 19.1: Discuss the implications of using data that were not created or collected to answer the specific research	Strengths/Limitations section, pp. 12–13<br><br>Limitations p. 21: administrative data noted; potential coding

		and magnitude of any potential bias		question(s). Include discussion of misclassification bias, unmeasured confounding, missing data, and changing eligibility over time, as they pertain to the study being reported.	inaccuracies, missing contextual variables, and residual linkage error acknowledged
Interpretation	20	Give a cautious overall interpretation of results considering objectives, limitations, multiplicity of analyses, results from similar studies, and other relevant evidence	Discussion and Conclusion		Discussion and Conclusion, pp. 18-21
Generalisability	21	Discuss the generalisability (external validity) of the study results	Discussion (Strengths and Limitations)		Limitations p. 21: generalisability beyond Australian context discussed; CALD underrepresentation noted
**Other Information**					
Funding	22	Give the source of funding and the role of the funders for the present study and, if applicable, for the original study on which the present article is based	Funding statement and Acknowledgement		Funding statement and Acknowledgements, p. 22
Accessibility of protocol, raw data, and programming code		..		RECORD 22.1: Authors should provide information on how to access any supplemental information such as the study protocol, raw data, or programming code.	Data Sharing statement. The manuscript states that the data used for the analysis will be made available upon reasonable request to the corresponding author. Supplementary material is provided with the manuscript.

*Reference: Benchimol EI, Smeeth L, Guttmann A, Harron K, Moher D, Petersen I, Sørensen HT, von Elm E, Langan SM, the RECORD Working Committee. The REporting of studies Conducted using Observational Routinely-collected health Data (RECORD) Statement. *PLoS Medicine* 2015; in press.

*Checklist is protected under Creative Commons Attribution (CC BY) license.

Supplementary Methods

Equation 1

$$Unplanned\ ED\ Presentation = \left(\frac{Discharged\ Home\ and\ Subsequent\ ED\ Presentation}{Total\ Records\ with\ Discharge\ Disposition\ of\ "Home"}\right) * 100$$

Equation 2

$$Planned\ ED\ Presentation = \left(\frac{Recommend\ ED\ and\ Subsequent\ ED\ Presentation}{Total\ Records\ with\ Discharge\ Disposition\ of\ "ED"}\right) * 100$$
